# Supplementary material for: Causal Relationship between Aging and Anorexia Nervosa: A White-Matter-Microstructure-Mediated Mendelian Randomization Analysis
Source: Biomedicines. 2024 Aug 16;12(8):1874. doi: 10.3390/biomedicines12081874 (PMC11351342; doi:10.3390/biomedicines12081874)
Supplement: Supplementary file 1 [file biomedicines-12-01874-s001.zip › Supplementary Figure/Figure S4/Singe SNP plot of MR analysis for TL to ubm-b-1610.pdf]

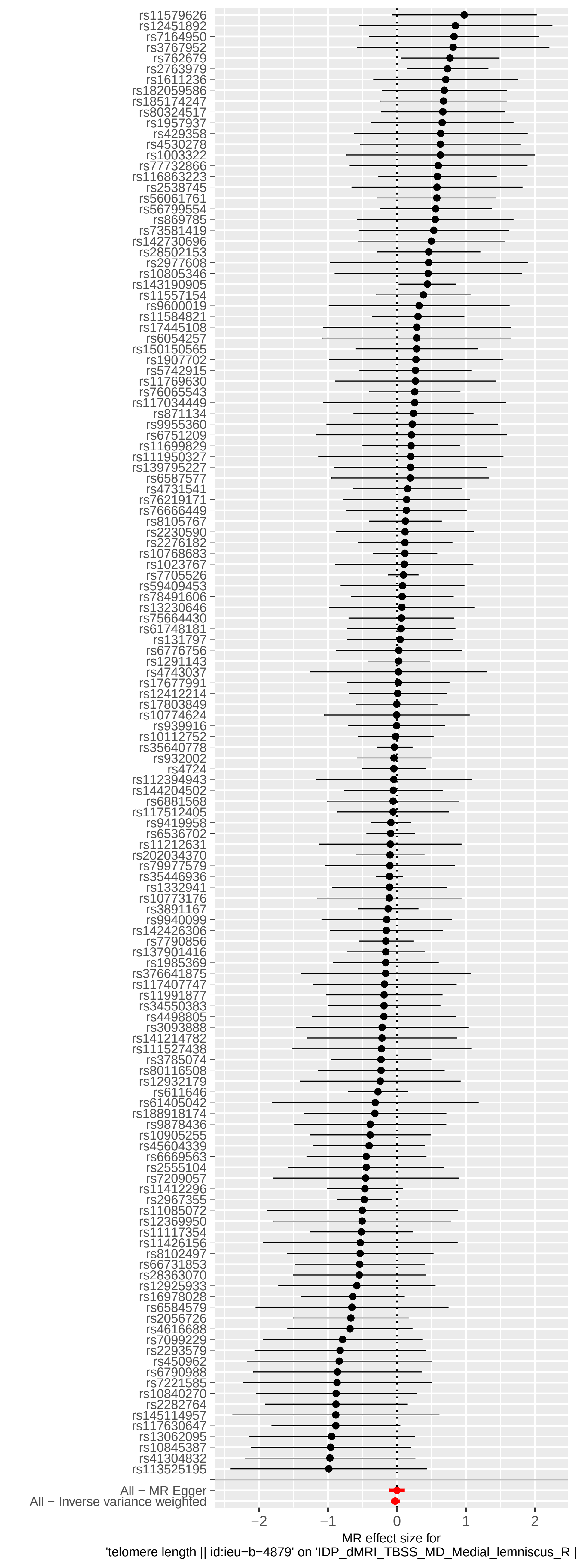

rs11579626
rs12451892
rs7164950
rs3767952
rs762679
rs2763979
rs1611236
rs182059586
rs185174247
rs80324517
rs1957937
rs429358
rs4530278
rs1003322
rs77732866
rs116863223
rs2538745
rs56061761
rs56799554
rs869785
rs73581419
rs142730696
rs28502153
rs2977608
rs10805346
rs143190905
rs11557154
rs9600019
rs11584821
rs17445108
rs6054257
rs150150565
rs1907702
rs5742915
rs11769630
rs76065543
rs117034449
rs871134
rs9955360
rs6751209
rs11699829
rs111950327
rs139795227
rs6587577
rs4731541
rs76219171
rs76666449
rs8105767
rs2230590
rs2276182
rs10768683
rs1023767
rs7705526
rs59409453
rs78491606
rs13230646
rs75664430
rs61748181
rs131797
rs6776756
rs1291143
rs4743037
rs17677991
rs12412214
rs17803849
rs10774624
rs939916
rs10112752
rs35640778
rs932002
rs4724
rs112394943
rs144204502
rs6881568
rs117512405
rs9419958
rs6536702
rs11212631
rs202034370
rs79977579
rs35446936
rs1332941
rs10773176
rs3891167
rs9940099
rs142426306
rs7790856
rs137901416
rs1985369
rs376641875
rs117407747
rs11991877
rs34550383
rs4498805
rs3093888
rs141214782
rs111527438
rs3785074
rs80116508
rs12932179
rs611646
rs61405042
rs188918174
rs9878436
rs10905255
rs45604339
rs6669563
rs2555104
rs7209057
rs11412296
rs2967355
rs11085072
rs12369950
rs11117354
rs11426156
rs8102497
rs66731853
rs28363070
rs12925933
rs16978028
rs6584579
rs2056726
rs4616688
rs7099229
rs2293579
rs450962
rs6790988
rs7221585
rs10840270
rs2282764
rs145114957
rs117630647
rs13062095
rs10845387
rs41304832
rs113525195
All − MR Egger
All − Inverse variance weighted
−2
−1
0
1
2
MR effect size for
'telomere length || id:ieu−b−4879' on 'IDP_dMRI_TBSS_MD_Medial_lemniscus_R |
